# Supplementary material for: Vitamin D Supplementation in the Czech Republic: Socioeconomic Determinants and Public Awareness Gaps
Source: Nutrients. 2025 Aug 13;17(16):2623. doi: 10.3390/nu17162623 (PMC12388922; doi:10.3390/nu17162623)
Supplement: Supplementary file 1 [file nutrients-17-02623-s001.zip › nutrients-3791142-supplementary.pdf]

Supplementary data:

Exploring Supplementation Trends and Knowledge of Vitamin D Effects in the Czech Republic

Questionnaire

1. Do you take a food supplement/drug that contains vitamin D?

1) Yes, I take it all year, regularly every day
2) Yes, I take it seasonally (periods with less sunshine), regularly every day
3) yes, I take it irregularly, when I remember, throughout the year
4) yes, I use irregularly, when I remember, during certain times of the year (seasonally)
5) no, I don't use ► go to question 4

QUESTIONS 2 AND 3 SHOULD BE ASKED ONLY TO THE RESPONDENTS WHO MARKED 1,2,3 OR 4 IN QUESTION 1.

2. When did you start taking vitamin D?

1) I started taking vitamin D it before the pandemic (before 2019).
2) I only started taking vitamin D it during the pandemic (2020-2021).
3) I only took vitamin D during the pandemic (2020-2021)
4) I started using vitamin D after the pandemic (2022)

3. Why are you taking vitamin D? (Multiple choices are possible.)

1) My doctor prescribed vitamin D due to a diagnosed vitamin D deficiency
2) My pharmacist recommended that I take vitamin D by my pharmacist
3) My family, friends, acquaintances
4) I am taking based on information from an advertisement for a particular vitamin D product (TV, radio, Internet, outdoor ads)
5) I use vitamin D based on an educational program that informs the importance of vitamin D
6) I use vitamin D at my own discretion

4. What processes in the body are affected by vitamin D? (Multiple choice is possible.)

1) Vitamin D helps maintain healthy bones (without it there is a higher risk of osteoporosis)

2) Vitamin D supports the proper functioning of the immune system

3) Vitamin D supports proper functioning of the cardiovascular system

4) Vitamin D worsens skin conditions (increases incidence of acne, eczema)

5) Vitamin D reduces the risk of metabolic diseases (obesity, diabetes, and metabolic syndrome)

6) Vitamin D helps maintain the health of the nervous system, including brain function

7) Vitamin D negatively affects lung health and functioning

8) Vitamin D increases the risk of allergies

9) Vitamin D increases the risk of most types of cancer

Table S1. Suitability of the model to detect dependencies between parameters

N = 1812	Analysis of deviance p value	R^2 model	% correctly clasified
Gender	0.000	0.12	38.02%
Age	0.002	0.07	40.1%
Place of residence	0.28	0.03	26.0%
Occupation	0.000	0.14	36.3%
Marital status	0.000	0.07	28.42%
Income	0.005	0.06	37%
Education	0.000	0.09	42.40%

Table S2 Regression analysis of vitamin D supplementation on socioeconomic factors, reference value never supplemented

Independent variable	Ans	RC	p value	OR	Lower Confidence Limit	Upper Confidence Limit
**Gender (reference male)**						
Female	1	0.90	0.001***	2.47	1.84	3.31
	2	0.60	0.001***	1.82	1.48	2.23
**Age (reference 15-19)**						
20-24	1	-0.31	0.56	0.74	0.26	2.06
	2	-0.07	0.80	0.93	0.53	1.64
25-34	1	0.33	0.41	1.39	0.63	3.04
	2	0.22	0.36	1.25	0.78	1.99
35-44	1	0.32	0.41	1.38	0.65	2.95
	2	0.03	0.91	1.03	0.65	1.62
45-54	1	0.33	0.39	1.39	0.65	2.94
	2	-0.06	0.80	0.94	0.6	1.49
55-64	1	0.44	0.26	1.56	0.73	3.35
	2	-0.18	0.47	0.84	0.52	1.35
≥65	1	0.91	0.01**	2.49	1.22	5.08
	2	-0.14	0.53	0.87	0.55	1.35
**Marital status (reference single)**						
Married	1	0.68	0.001***	1.97	1.34	2.88
	2	-0.14	0.24	0.87	0.68	1.1
Divorced	1	0.28	0.31	1.32	0.77	2.28
	2	-0.22	0.21	0.8	0.57	1.13
Widower, Widow	1	0.98	0.001***	2.68	1.61	4.46

	2	-0.21	0.30	0.81	0.55	1.2
Partner, Common-law Partner	1	-0.04	0.93	0.96	0.38	2.41
	2	-0.02	0.93	0.98	0.58	1.63
**Education (reference Primary/Elementary)**						
Vocational Training or Secondary Education without Diploma	1	0	0.99	1	0.52	1.93
		-0.28	0.21	0.75	0.48	1.17
High School Diploma, Higher Vocational Education	1	0.37	0.25	1.45	0.77	2.72
		0.39	0.07	1.47	0.97	2.24
Bachelor’s Degree, University Degree	1	0.78	0.02*	2.18	1.13	4.2
	2	0.44	0.06	1.55	0.99	2.43
**Income (reference 0 – 20,000)**						
20,001 – 30,000	1	0.33	0.29	1.38	0.76	2.53
	2	-0.04	0.86	0.96	0.60	1.54
30,001 – 40,000	1	-0.23	0.46	0.79	0.43	1.47
	2	0.10	0.66	1.11	0.70	1.74
40,001 – 50,000	1	-0.20	0.53	0.81	0.43	1.54
	2	0.25	0.28	1.29	0.81	2.04
50,001 – 60,000	1	0.17	0.59	1.19	0.63	2.23
	2	0.40	0.1	1.49	0.93	2.37
60,001 – 70,000	1	-0.05	0.90	0.95	0.47	1.93
	2	0.62	0.01**	1.87	1.15	3.04
≥ 70,001	1	0.26	0.41	1.30	0.70	2.43
	2	0.28	0.25	1.32	0.82	2.11
**Occupation (Manager, Director, Executive)**						
2	1	0.39	0.31	1.47	0.70	3.09
	2	0.79	0.01	2.2	1.17	4.12
3	1	-0.18	0.63	0.83	0.40	1.74
	2	0.42	0.17	1.52	0.83	2.76
4	1	-0.48	0.15	0.62	0.32	1.19
	2	0.36	0.19	1.43	0.84	2.45
5	1	0.02	0.94	1.02	0.56	1.89
	2	0.04	0.89	1.04	0.61	1.77
6	1	-1.22	0.02*	0.29	0.11	0.81
	2	-0.41	0.26	0.67	0.33	1.35
7	1	-0.71	0.05*	0.49	0.24	1.01
	2	0.35	0.22	1.42	0.81	2.48
8	1	-0.88	0.03*	0.41	0.18	0.93
	2	0.30	0.33	1.34	0.75	2.42
9	1	-1.14	0.003**	0.32	0.15	0.69
	2	-0.21	0.49	0.81	0.45	1.45

OCCUPATION: 1 Manager, Director, Executive, 2 Mental Worker (Scientist, Doctor, Teacher, Priest, Actor, etc.; 3 Clerk, Administrative Worker; 4 Service Worker (Salesperson, Sales Representative, Nurse, etc.); 5 Retiree, Pensioner, Disabled Pensioner; 6 Self-employed (with up to 3 employees); Entrepreneur (employing more than 3 employees); 7 Student; Homemaker, Parental leave; Unemployed; 8 Armed Forces Employee (Soldier, Police Officer, Guard, Security Service); Engineer, Technician; 9 Farmer, Agricultural Worker, Forester; Labourer, Worker; Craftsman (without own business)

RC: regression coefficient; OR: odds ration; Ans: answers; Ans 1: regular supplementation; Ans 2: irregular supplementation

R: reference group

Table S3 Numbers of respondents in each group according to vitamin D supplementation

	Use regularly	Non-use	Use irregularly	Total
Marital status				
Single	41	256	195	492
Married	137	435	287	859
Divorced	25	118	72	215
Widower, Widow	36	84	52	172
Partner, Common-law Partner	6	39	29	74
Education				
Primary/ Elementary	13	69	39	121
Vocational Training or Secondary Education without Diploma	60	317	135	512
High School Diploma, Higher Vocational Education 3	103	378	314	795
Bachelor’s Degree, University Degree	69	168	147	384
Income				
10,001 – 20,000	18	72	39	129
20,001 – 30,000	54	156	81	291
30,001 – 40,000	39	197	118	354
40,001 – 50,000	33	162	113	308
50,001 – 60,000	38	128	103	269
60,001 – 70,000	21	88	89	198
70,001 – 80,000	42	129	92	263
Occupation				
Manager, Director, Executive	17	45	25	87
Mental Worker	25	45	55	125
Clerk, Administrative Worker	22	70	59	151
Service Worker	41	176	140	357
Retiree, Pensioner, Disabled Pensioner	84	217	125	426
Self-employed, entrepreneur	6	54	20	80

Student, Homemaker, Parental leave, Unemployed = without salary	22	118	93	233
Armed Forces Employee, engineer, technician	13	83	62	158
Farmer, Agricultural Worker, Forester, Labourer, Worker, Craftsman (without own business)	15	124	56	195
